# Characteristics of Lyme optic neuritis: a case report of Lyme associated bilateral optic neuritis and systematic review of the literature

**DOI:** 10.1186/s12883-022-02627-z

**Published:** 2022-03-23

**Authors:** Yezhong Lu, Ramin Zand

**Affiliations:** 1 Geisinger Commonwealth School of Medicine, Scranton, PA USA; 2 Department of Neurology, Neuroscience Institute, Geisinger Health System, Danville, PA USA

## Abstract

Optic Neuritis is rare in Lyme borreliosis. The current knowledge of optic nerve involvement in Lyme borreliosis relies solely on case reports. The aim of this systematic review was to characterize and investigate the associated factors of optic neuritis in Lyme borreliosis. We further presented a very rare case of isolated bilateral optic neuritis in a Lyme seropositive patient.

## Background

Lyme borreliosis, caused by *Borrelia burgdorferi*, is the most frequent reported vector-borne disease in the United States [1, 2]. Steere et al., (1977) first introduced clinical characteristics of Lyme borreliosis as recurrent, asymmetric, short attacking arthritis, and often precede in skin lesions [3]. Lyme borreliosis can affect multiple systems and has various manifestations that occur in stages. The clinical course of Lyme borreliosis begins with skin lesions [4]. Neurological, cardiac, musculoskeletal and rheumatological presentation usually develop in 2nd and 3rd stage of the disease [2, 5–7]. However, presentations in each chronological stage has not been always consistent [8].

Kauffmann & Wormser (1990) was first to describe a case which the uniocular uveitis progressed to panedopthalmitis and loss of vision due to Lyme borreliosis [9]**.** Ocular involvement is usually seen in the 2nd or 3rd stage of the disease. Although relatively uncommon, it could manifest multifariously such as conjunctivitis, keratitis, uveitis, periorbital oedema, cranial nerve II, III, IV, VII palsies, papilledema, reversible Horner’s syndrome, cotton wool spot, vascular occlusion, and optic neuritis [9–17]. Optic neuritis is rare in Lyme borreliosis; therefore, it is often overlooked in the differential diagnosis.

The goal of this systematic review was to characterize and investigate the associated factors of optic neuritis in Lyme borreliosis. We further presented a very rare case of isolated bilateral optic neuritis in a Lyme seropositive patient.

## Methodology

A systemic review of Lyme optic neuritis cases characteristics was performed according to the preferred reporting items for systematic reviews and meta-analysis (PRISMA) statement. The electronic database Google Scholar was the primary source for article identification and PubMed was used for supplement. Articles were searched from database inception to July 2021 and identified through Keywords “Optic Neuritis”, paired with “Lyme disease”, “Lyme Borreliosis”, “Case report” and “Erythema Migrans”. MeSH term “Lyme Disease” paired with “Optic Neuritis” was used for search in PubMed. Accessible articles in English language were appraised and assessed via case report guidelines (CARE) by one individual and reference lists were scanned for additional studies of potential relevance.

Articles that include elements of clinical assessment/diagnosis of optic neuritis and Lyme borreliosis (positive 2 tier serology tests), therapeutic interventions and outcome were included. Demographics, clinical findings, treatment, and treatment outcomes are listed in Table 1 and Table 2.

**Table 1:** The reported cases of Lyme Optic neuritis

Cases	Age/Sex	Tick Bite	Erythema Migrans	Signs and Symptoms	Fundus	Imaging/Testing	Treatment	Treatment outcome
Cruz, et al. (2020) [18]	48/M	Does not recall	Unreported	1. Blurry vision OD 2. Inferior visual field defect 3. RAPD OD 4. Photopsia	1. Pale optic disc 2. Edema of inferior quadrant OD 3. Hyperemia and diffuse edema OS	**MRI**: unremarkable **VEP**: delayed bilaterally **VF**: generalized deficit on the right eye and a left inferior scotoma	Ceftriaxone	Improved, lost to follow up due to patient relocation
Jha, et al. (2018) [19]	46/F	Does not recall	Unreported	1. Progressive blurry vision > 3 wks. 2. Upper respiratory symptoms 3. Paresthesia 4. Nausea 5. Weakness 6. Bilateral lower extremities paresthesia	1. Bilateral optic head edema 2. Hyperemia 3. Optic disc edema	**MRI**: nonspecific white matter hyperintensities **VF**: Cecocentral defects OU	Doxycycline	Patient did not return for follow up
Wang, et al.(2017) [20]	50/F	Does not recall	Pruritic rash on neck, chest, and right lower extremities	1. Right sided headache 2. Unspecified eye pain 3. Blurry vision OD	1. Optic head edema OD	**MRI**: enhancement of right optic nerve	Ceftriaxone Methylprednisolone	Improved in symptoms MRI shows resolution of findings
Burakgazi and Henderson (2016) [21]	59/F	Does not recall	Shoulders extending to midback, part of chest, left shoulder and right cheek	1. Blurry vision OD for 3–4 wks. 2. Fatigue and generalized joint pain	1. Optic head edema OD	**MRI**: unremarkable **VF**: unspecified visual field defect **VA**: 20/30 OD, 20/20 OS	Doxycycline	Improved visual deficits and symptoms
Tzoukeva, et al. (2014) [22]	42/F	Does not recall	Absence	1. Progressive blurry vision OS 2. Painful ocular movement 3. Left RAPD 4. Decreased color vision	Unremarkable	**MRI**: typical lesion characteristics of MS	Methylprednisolone Medaxone Cefprozil	Normalized
Qureshi, et al. (2016) [23]	43/M	Does not recall	unreported	1. Headache 2. Paresthesia 3. Nuchal rigidity 4. Kernig and Brudzinski sign 5. Seizure episodes	1. Posterior uveitis 2. Bilateral papillitis	**MRI**: diffuse hyperintensities involving supra and infratemporal cortical sulci consistent with diffuse leptomeningoencephalitis	Doxycycline Ketorolac	Normalized
McVeigh and Vakros (2012) [24]	48/M	Denies recent bites, but had sustained bites previously	Absence	1. Bilateral loss of visual acuity 2. Painful ocular movements 3. Photophobia 4. Visual distortion 5. Ataxia 6. Headache	1. Uveitis 2. Bilateral disc swelling 3. Splinter hemorrhage OD	Unspecified	Oral Steroid Ceftriaxone Doxycycline Amoxicillin	Normalized (Mild pallor of the left disc)
Blanc, et al.(2010) [25]	63/F	Yes	Unreported	1. Blurry vision OD 2. Headache 3. Arthralgia	Unspecified	**MRI**: 3 nonspecific lesions on T2 FLAIR and T2 weighted **VEP**: delayed bilaterally **VA**: 20/200 OD	Ceftriaxone	Visual acuity improved to 20/80
Blanc, et al.(2010) [25]	48/F	Yes	Denies	1. Arthralgia 2. Headache 3. Blurry vision	1. Bilateral papillitis	**MRI**: 2 nonspecific subcortical Flair lesions in left frontal lobe (< 3 mm in diameter) **VA**: 20/100 OS	Ceftriaxone Methylprednisolone	Visual acuity improved to 20/20
Santino, et al. (2009) [26]	33/M	Unspecified	Unspecified	1. Right central scotoma 2. Blurry vision	1. Bilateral papillitis	**VA**: 4/10 OD 10/10 OS	Methylprednisolone Ceftriaxone	Visual acuity improved to 10/10 OU
Krim, et al. (2007) [27]	67/M	Yes	Right arm	1. Fatigue 2. Myalgia 3. Neck radiculopathy 4. Facial weakness 5. Ptosis 6. Diplopia 7. Fever 8. Peripheral right facial palsy 9. Right arm paresis 10. Retrobulbar pain 11. Diminished color perception OD	1. Bilateral uveitis	**MRI**: unremarkable **VEP:** delayed OD **VF**: central scotoma OD **VA**: 5/10 OD 8/10 OS	Ceftriaxone Corticotherapy	Neurological symptoms resolved, and visual acuity improved to 10/10 OU

*MRI* Magnetic resonance imaging, *VA* Visual acuity, *VEP* Visual evoked potential, *VF* Visual field

**Table 2:** Cerebrospinal Fluid (CSF) Analysis of the reported cases

Cases	CSF protein	CSF cell count
Cruz, et al. (2020) [18]	Elevated (185.4 mg/dL)	Elevated white cell count (177 cells/mL) lymphocytic predominance
Jha, et al. (2018) [19]	Normal	Normal
Wang, et al.(2017) [20]	Normal	Elevated (20 red cells, 2 white cells)
Burakgazi and Henderson (2016) [21]	Elevated (55 mg/dL)	Normal
Tzoukeva, et al. (2014) [22]	Not performed	Not performed
Qureshi, et al. (2016) [23]	Elevated (94 mg/dL)	Elevated white cell counts (288 white cells) lymphocytic predominance (98%)
McVeigh and Vakros (2012) [24]	Unreported	Elevated
Blanc, et al.(2010) [25]	Elevated (0.49 g/l)	Normal
Blanc, et al.(2010) [25]	Elevated (0.64 g/l)	Normal
Santino, et al. (2009) [26]	Elevated	Elevated white cell count lymphocytic predominance
Krim, et al. (2007) [27]	Elevated (1.11 g/l)	Elevated white cell count (21 /mm^3^) lymphocytic predominance (95%)

We also presented a very rare case of isolated bilateral optic neuritis in a Lyme seropositive patient. The written consent was obtained from the patient to present her illness.

## Case presentation

A 48 years old female with the past medical history of multiple sclerosis (MS), presented to her primary care physician in December 2019, with fever and sore throat. Three weeks later, patient returned and reported development of photophobia, eye pressure sensation, blurry vision, pain with eye movements for more than 3 weeks and noted central scotoma during the morning prior to her visit. Patient’s MS was first diagnosed in 2006. She has had relapses in 2010 and 2018, which both mainly presented as fatigue and difficulties in walking. Patient was on Copaxone (2006–2009), Gilenya (2010–2018) and currently treated with Ocrelizumab.

**Fig. 1:**
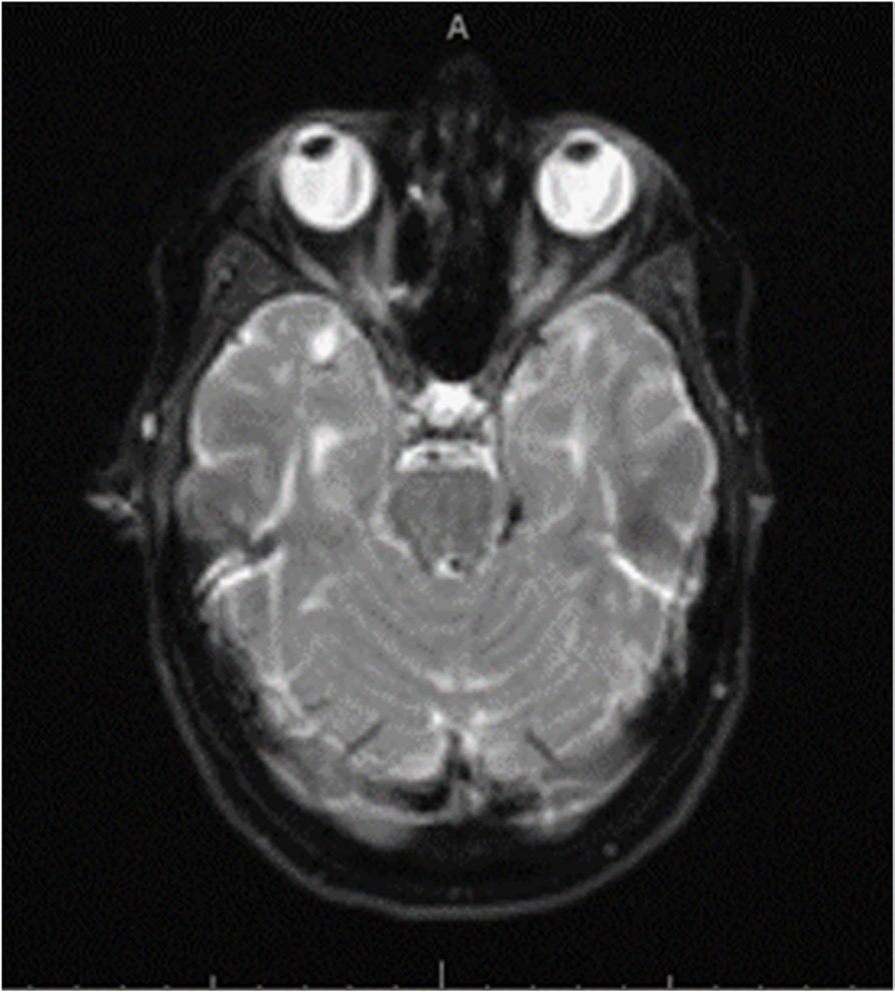
Optical coherence tomography revealed bilateral optic disc edema and peripapillary retina nerve fiber layer thickening

**Fig. 2:**
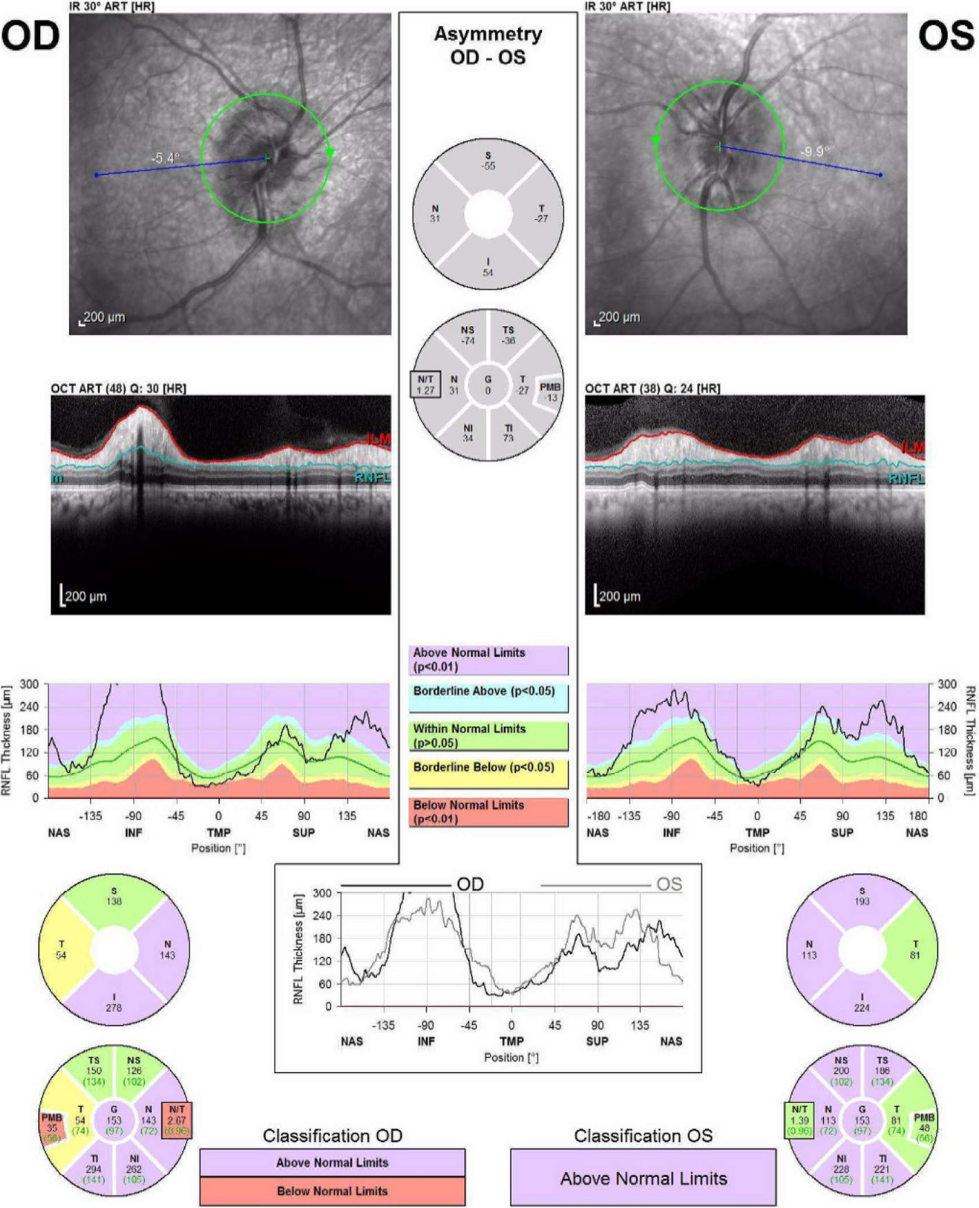
MRI of the orbits shows thickened optic nerve with increased T2 signal and marked enhancement

She did not report any new neurological deficit except blurry vision. Her fundus exam and optical coherence tomography (OCT) revealed bilateral disc edema, and peripapillary retina nerve fiber layer thickening OU (Fig. 1). Her visual acuity (Snellen linear chart) was 20/40 OU, intraocular pressures (non-contact) were 19 (mm Hg) OD and 18 (mm Hg) OS, Ishihara test resulted 8/8 OD and 7/8 OS, confrontational visual field full, and pupils equal round and reactive to light. Magnetic resonance imaging (MRI) of the orbit showed thickened and increased T2 signal of the optic nerve. MRI along with fundus exam confirmed the diagnosis of bilateral optic neuritis (Fig. 2). MRI of the brain showed similar burden of supratentorial and infratentorial T2/Fluid-attenuated inversion recovery (FLAIR) hyperintensities lesions compatible with her known MS, and no new lesions identified. Anti- Borrelia IgM were both positive in serum and cerebrospinal fluid (CSF) and was confirmed by western blotting. Patient admitted her husband removed a tick from her leg 2-month prior to her visit but denied any rashes.

CSF analysis revealed mononuclear pleocytosis, and elevated glucose. CSF flow cytometry showed 27% neutrophils, 40% lymphocytes and 33% monocytes (Table 3). Patient was admitted to the hospital for 4 days, treated with intravenous methylprednisolone (1 g/day for 3 days) immediately given the degree of swelling, then she was administered ceftriaxone (2 g/day for 25 days) for the management of Lyme borreliosis. Serum myelin oligodendrocyte glycoprotein fluorescence-activated cell sorting assay (MOG FACS) and Neuromyelitis Optica fluorescence-activated cell sorting assay (NMO FACS) were sent to mayo laboratories and returned negative.

**Table 3:** Summary of findings in the patient

	Serum IgG/IgM Elisa	Serum IgG WB bands (kD)	Serum IgM western blot bands (kD)	CSF Glucose (mg/dL)	CSF protein (mg/dL)	_CSF WBC (cells/mm_^3^_)_	_CSF RBC (cells/mm_^3^_)_	CSF IgG Bands (kD)	CSF IgM Bands (kD)
Patient	6.9	23,39,41,93	23,39,41	75	133	275	4	3, 41, 93	23, 39
Normal Value	< 0.91	Negative If not detected or fewer than 5 of 10 significant bands	Negative if not detected or less than 2 bands.	45–70	15–45	0	0	–	–

Patient reported significant improvement of her visual problems after she finished her corticosteroid therapy. Patient returned for follow up 1 week post hospitalization, reported visual symptoms abated and she was back to her previous baseline. Patient’s prior most recent ophthalmology visit was in April 2013, her fundus exam revealed bilateral temporal disc pallor. Best corrected vision (Snellen linear chart) was 20/20 OD, 20/25 OS, color vision, visual field and extraocular movement were all full. There was no signs or symptoms of optic neuritis.

## Review of literature

**Fig. 3:**
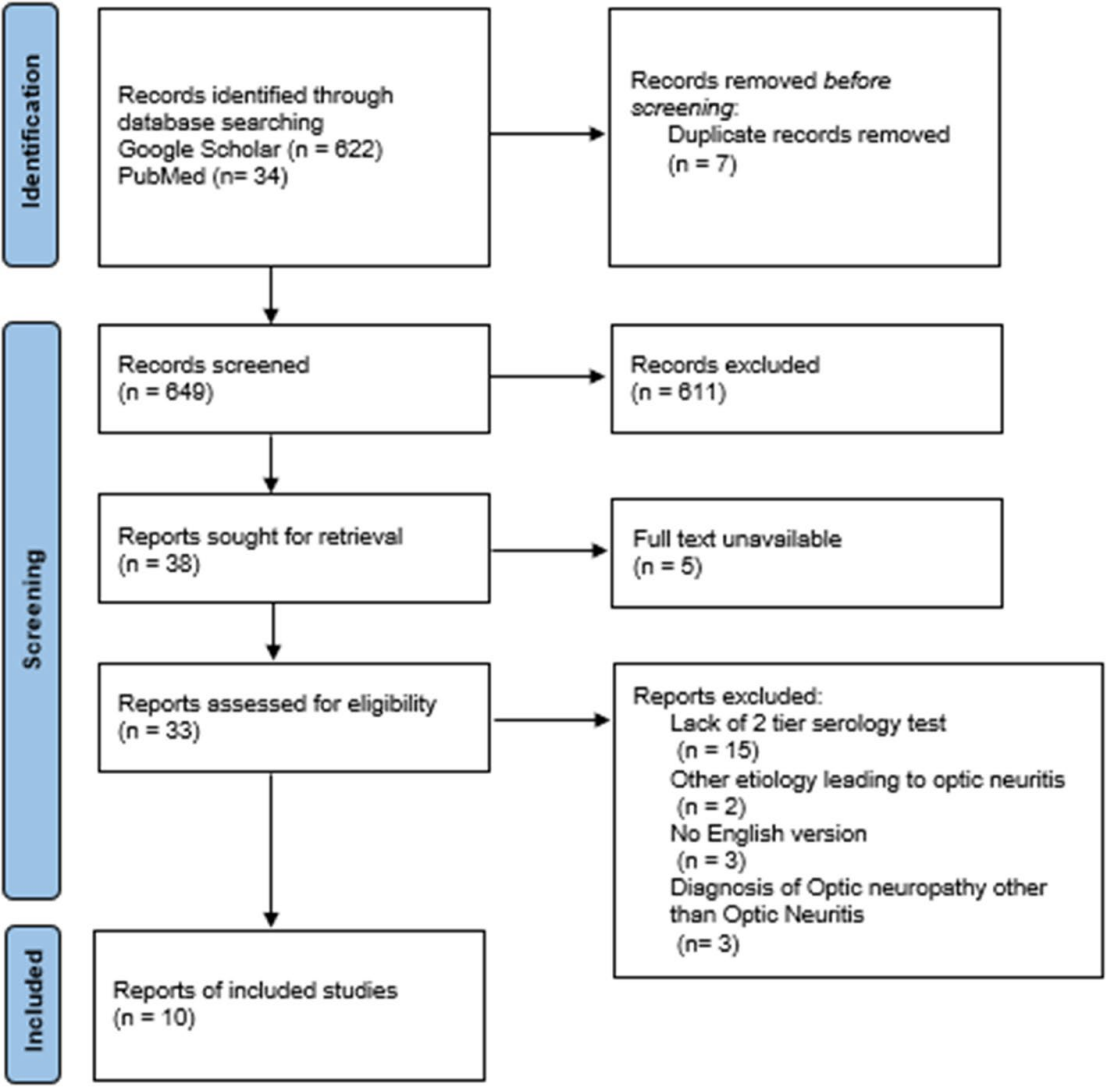
Flow chart of search strategy

A total of 656 reports were retrieved by searching through Google Scholar and PubMed. After removing the duplicates and adding the manually tracked citations, 649 titles and abstracts were screened. Among the reviewed full texts, 33 reports were candidates of being included in this review and assessed for eligibility. We excluded 23 reports due to lack of confirmatory positive western blot following a positive ELISA test (15 reports), other etiology leading to optic neuritis (2 reports), no English version of the text (3 reports), and diagnosis of optic neuropathy other than optic neuritis (3 reports) (see Fig. 3). Total of 10 reports and 11 patients with optic neuritis and Lyme borreliosis were included in this review [18–27].

The patients’ age ranged from 33 to 67 years (median 48 years), 5 were male and 6 were female. Seven cases were from Europe and 5 were from North America. The most common symptoms reported are related to optic neuritis – blurry vision (11 cases), headache (7 cases), scotoma (3 cases) and painful ocular movement (3 cases). Besides visual complaints, 4 reported neurological symptoms – paresthesia (3 cases) and ataxia (1 case); 3 reported arthralgia; and 3 reported nonspecific symptoms – fatigue, weakness, and myalgia.

The most common signs found are bilateral optic disc edema (8 cases) and relative afferent pupillary defect (2 cases). Erythema migrans was diagnosed in 2 of the total patients. Eight patients did not recall having tick bites. Moderate vision loss (better than 20/200) was observed with majority of the patients (9 cases).

Ten out of 11 patients have CSF study (see Table 2). They all revealed a normal opening pressure and glucose level. Common laboratory findings were elevated cerebral spinal fluid protein levels (6 cases), and mononuclear pleocytosis (4 cases).

The patients all responded well with combination of corticosteroid and antibiotic therapy, or antibiotic therapy alone. Of the 5 patients treated with solely antibiotic therapy, except 2 who did not return for follow up, the rest showed improvements or resolution of symptoms. The 6 patients who received combination therapy also showed improvements or normalization of the symptoms.

## Discussion

Optic Neuritis has been reported in both the US and Europe in patients with neuroborreliosis or positive Lyme serologies, however the relationship remains elusive due to insufficient knowledge and multiple confounding variables [12, 28–30]. Majority of the cases in the literature showed compelling clinical signs of Lyme borreliosis, however, did not meet the confirmatory diagnosis criteria according to Centers for Disease Control and Prevention (CDC) [31]. The relationship between Lyme borreliosis and optic neuritis has been controversial. In a retrospective study, Sibony et al., 2005, reported prevalence of 4% of optic neuritis patients with positive Lyme serology was possibly secondary to Lyme borreliosis [32]. But in another cohort study of 81 patients with neuroborreliosis, 27% reported to have delayed visual evoked potential, which suggests that prevalence of visual involvement in Lyme disease could be higher [33, 34].

Diagnosis of Lyme borreliosis is established based on clinical presentation with supportive findings from laboratory testing [31]. Laboratory diagnosis of Lyme borreliosis is established through enzyme-linked immunosorbent assay (ELISA) for IgM and IgG antibodies against *Borrelia burgdorferi*. In patient with positive ELISA response, western blots were performed to confirm the specificity of the antibodies [31]. Immunoblot requires at least 2 of 3 signature bands (23 kDa, 39 kDa, 41 kDa) for a positive IgM and at least 5 of 10 signature bands (18 kDa, 23 kDa, 28 kDa, 30 kDa, 39 kDa, 41 kDa, 45 kDa, 58 kDa, 66 kDa, 93 kDa) for a positive IgG [35].

The diagnosis of Lyme borreliosis remains challenging. Serology tests results are frequently misinterpreted leading to misdiagnosis and can lead to serious morbidity. Despite having high sensitivity, ELISA results could be confounded by delayed immune response, false positivity, and high prevalence of asymptomatic seropositivity in endemic areas [4, 32, 36, 37]. In addition, the diagnosis is made difficult by long incubation period and symptoms mimicking a wide range of disease processes, such as fibromyalgia, syphilis, Alzheimer’s and autoimmune disorders [36–41]. Due to the limitations, Lyme borreliosis is frequently misdiagnosed or delayed in diagnosis. The CDC reports 30,000 cases of Lyme borreliosis annually from 2008 to 2014, but estimates true incidence is much higher [42]. The public health burden of Lyme borreliosis continues to grow substantially each year [43]. It is crucial for clinicians working in endemic regions to be aware and recognize of signs and symptoms of Lyme borreliosis.

The pathophysiology of Lyme borreliosis in various organs at different stages remains controversial due to infrequency of finding of *Borrelia burgdorferi* via direct testing [41, 44]. *Borrelia burgdorferi* has been successfully cultured from various tissues, like blood and synovial fluid, and also immune privileged sites like the eyes and brain, but the mechanism of entry remains unclear [9, 45, 46]. Current evidence suggests pathogenesis in the central nervous system is via direct cytotoxicity, neurotropism and production of neurotoxic and proinflammatory mediators [47–53]. Unlike other bacterial infections which elicit neutrophil infiltration in the CSF, Borrelia species produce lymphocytic pleocytosis and enhanced intrathecal antibody production [54, 55]. Optic nerve involvement in Lyme borreliosis has been rare and causal relationship has been difficult to prove. Currently, there is no clinical guidelines as when Lyme borreliosis should be considered in optic neuritis.

In this review, we collected cases that have demonstrated strong evidence of causal relationship of Lyme borreliosis and optic neuritis in attempt to characterize the nature and clinical presentations of optic neuritis involved in Lyme borreliosis. Importantly, there are few limitations and concerns need to be highlighted. Despite all the cases collected in this review having positive 2 tier Lyme serology (Table 1), majority of cases could still remain idiopathic (absence of tick bites and erythema migrans); the cause of the symptoms could be associated with undiagnosed underlying demyelinating conditions such as multiple sclerosis which will require a long term follow up to establish the diagnoses [56]. Additionally, 4 of 10 cases with CSF analysis revealed normal CSF cell count which led to questions of whether there are other underlying etiologies. Regardless of the differences and limitations, there are few pertinent features that deserves considerations. Majority of the cases present with features of atypical optic neuritis that deviate from the characteristics of typical idiopathic demyelinating optic neuritis. Typical optic neuritis commonly presents with acute, painful, and self-limiting unilateral visual loss [57–59]. Our findings conclude Lyme optic neuritis usually presents with bilateral optic nerve head swellings, and painless, moderate (better than 20/200) and progressive visual loss. Common CSF analysis reveals elevated protein and mononuclear pleocytosis. These atypical features may provide a clue, however attention to presentations, detailed history taking, and correct interpretation of lab values are paramount for making the correct diagnosis and preventing future implications.

Additionally, our results indicate that these patients respond well to antibiotics and have good prognosis. Antibiotic therapy (14 to 21 day course) has been shown to be effective in treating Lyme borreliosis [60]. Antibiotics usually include doxycycline for adults, and amoxicillin or cefuroxime for adults, children, pregnant or breast feeding women [7]. Systemic corticosteroid without concomitant antibiotics should not be used in treatment of ocular Lyme disease [7]. For all antibiotics regimen, treatment failures and relapses have been reported, prolonged courses of therapy are not recommended. For treatment failures, underlying diagnosis should be reconsidered [38].

Finally, we present a case demonstrated a strong causal relationship of optic neuritis and Lyme borreliosis. Our patient’s optic neuritis could be reflective of her diagnosis of relapsing remitting multiple sclerosis. However, her presented symptoms were atypical for the patient’s MS due to absence of other neurological symptoms, and atypical compared to her flare ups in the past. Additionally, optic neuritis from multiple sclerosis is usually unilateral with normal or mild pupillary disc edema [40, 61, 62]. Initially, it was suspected that her condition was secondary to alternative inflammatory process such as neuromyelitis optica or myelin oligodendrocyte glycoprotein antibody demyelination due to bilateral involvement of her optic discs, but was later on ruled out by laboratory work up [63, 64]. CSF lymphocytic pleocytosis in the absence of meningeal signs, along with recent finding of ticks, positive serum Lyme antibodies and confirmatory test for Lyme borreliosis suggest her optic neuritis was secondary to Lyme borreliosis. Patient was administered IV ceftriaxone for the management of Lyme borreliosis, and steroid was given due to the degree of the swelling. Patient’s symptoms normalized after her treatments.

## Conclusion

Clinicians working in the endemic areas should consider Lyme borreliosis in patients presents with bilateral optic nerve head swelling, and painless progressive visual loss. Inadequate early treatment of Lyme borreliosis increases the likelihood of late manifestation and leads to relapses. Lyme borreliosis patients with optic neuritis respond well to antibiotics and have good prognosis.

## Data Availability

Data can be shared with other investigators upon request and execution of a data-sharing agreement.
